# Editorial: Understanding the impact of social media on public mental health and crisis management during the COVID-19 pandemic

**DOI:** 10.3389/fpsyg.2023.1304586

**Published:** 2023-10-16

**Authors:** Meng Cai, Pan Liu, Chengwei Xu, Han Luo

**Affiliations:** ^1^School of Humanities and Social Sciences, Xi'an Jiaotong University, Xi'an, China; ^2^School of Public Administration, Hunan University, Changsha, China; ^3^Public Management and Policy Analysis Program, Graduate School of International Relations, International University of Japan, Minamiuonuma, Japan

**Keywords:** network dissemination, social media, sentiment analysis, public crisis management, public mental health

The advent of the digital age has led to changes in the manner and nature of information generation, circulation, and reception. For example, social media connects people through text, pictures, and videos to build a vast social network (De Paor and Heravi, 2020) and significantly influence people's mindset and behaviors (Liu et al., 2018). At the same time, the efficiency of instant information exchange makes social media essential for risk management and responses to hazards, emergencies, and crises (Brengarth and Mujkic, 2016). During the time of crisis, information about the crisis could spread quickly beyond traditional media (e.g., newspapers and television) to social media platforms, including Twitter, Facebook, WhatsApp, WeChat, and Weibo, at an even more alarming speed (Martínez-Rojas et al., 2018). However, convenient information sharing and fast spreading are usually accompanied by misinformation. The lack of online regulation and the anonymity of users also provide a breeding ground for misinformation, disinformation, social bots, fake news, and rumors. The prevalence of misinformation/disinformation has significantly undermined the full benefits of social media use and brought many challenges to cyberspace security and social order. This could create a hostile internet environment where toxic public opinions, fear, anger, and hate spread quickly (Zhang et al., 2022), which would bring significant obstacles for governments in managing emergencies. Therefore, to understand the public responses and attitudinal dynamics and to promote more effective emergency management during emergencies, this Research Topic examines how social media use impacts people's mental health and government responses to crisis using several studies during the COVID-19 pandemic. The COVID-19 pandemic has damaged the world economy badly and taken millions of lives in the past 3 years. Studies in this Research Topic are committed to providing fresh evidence and theoretical insights for understanding human mental health and promoting effective crisis management.

During the COVID-19 outbreak, social media has become the essential communication method for isolated people at home, given the concern that the virus could be transmitted quickly in meetings and social gatherings. Information related to COVID-19, medicines, vaccines, and government response measures became the central topic in cyberspace. For example, governments disseminated policy suggestions for people to stay safe and promoted implementing some stringent measures using social media platforms. Besides, social media has been the main channel for isolated individual to seek social support, which is essential for mental and physical health (Straughan and Xu, 2023). The public relied on social media to keep in touch with their friends and relatives, learn how to protect themselves, share views and attitudes, and support one another. The diffusion of all kinds of information (scientific and lay knowledge) on social media highly influences recipients' attitudes, emotions and health practices (Straughan and Xu, 2022). Different knowledge, attitudes, and practices were shared and discussed online, which provides an effective channel for researchers and policymakers to understand the status quo of public mental health and public opinions (Liu et al., 2020). At the same time, during the pandemic, how to use the private data collected and stored on social media for the public's good is a critical issue. Researchers suggest that the big data collected on social media is helpful in allocating resources and providing support to fight against the pandemic and save lives (Cai et al., 2022). Studies of the present Research Topic draw together empirical studies that explore how social media use affects public mental health and emergency management in crisis. Papers on this Research Topic contribute to understanding people's responses, coping with negative emotions, and network conflicts. Beyond the knowledge contribution, researchers also discussed policy implications.

There are five articles in this Research Topic. The topics covered in the present Research Topic include network distribution, social media, sentiment analysis, and public crisis management. More specifically, the papers investigated public reactions and emotional dynamics during the COVID-19 pandemic, people's mental health under lockdown conditions, and the impact of social media on social support and public trust. Moreover, the present Research Topic also focused on comparing the impact of social media and traditional media on users' knowledge, attitudes, and health practices and the impact of social media on vaccine acceptance. The five articles examined the main theme of this Research Topic from different perspectives and evidence. We briefly introduce the articles in the next section. The articles are discussed according to the order in which they were published.

Article 1 (Ma et al.) collected users' interaction data from the Weibo platform. It examined the multidimensional attributes and characteristics of public sentiment during the outbreak of COVID-19 to meet the increasing demand for public opinion management on social media. The authors combined deep learning, topic clustering, and correlation analysis, and based on large-scale posts and comments, quantitatively analyzed the evolution law of public opinion in time series during the epidemic period and explored the content-based characteristics and audience response characteristics. Specifically, through the analysis of public opinion in the time series, the authors found that the trend of negative public opinion was divided internally, and there were specific window periods. The analysis of the content characteristics of public opinion showed that negative public opinion was more likely to cause public discussion with a larger scale and deep participation. Their analysis of the characteristics of audience response during the period of public opinion demonstrated that the generation of public opinion was independent of posts and users, and the guiding role of opinion leaders was limited. Finding in this paper provides insights for elevating the capacity of public opinion management, effective intervention, and improving the public opinion management system.

Article 2 (Sun et al.) focused on the mental health of individuals during the global pandemic. It analyzed how the stressors related to COVID-19 and the use of social media promoted the psychological adjustment of individuals' coping strategies based on the transactional model of stress and coping. The authors collected survey data from 641 quarantined residents during the COVID-19 pandemic to analyze the relationships between perceived COVID-19 stress, social media use, and personal coping strategies. Research results revealed the critical role of social media use in coping with stress and psychological adjustment, which could help readers to understand how social media use affected the mental health of individuals under public health crises as well as the impact of different ways of social media use on individual psychological adjustment.

Article 3 (Steinberger and Kim) investigated unhealthy user behaviors represented by social network addiction and looked into the relationship between subjective wellbeing and social network addiction through a cross-sectional survey. In this article, the authors examined two possible mediating factors between subjective wellbeing and social network addiction: social comparison and the fear of missing out. Social comparison was divided into two aspects: social comparison of ability and social comparison of opinion. The former related to the social outcomes described in social network posts (such as material wealth, health status, and personal achievements), and the latter referred to the beliefs and values expressed in the posts. Results showed that social comparison and the fear of missing out jointly mediated the relationship between subjective wellbeing and social network addiction, and social comparison of ability played a more important role in social network addiction than social comparison of opinion. In addition, the fear of missing out in social networks also relies more on the process of ability than opinion comparisons. The findings of this study offered support for understanding social network addiction and promoting the formation of healthy behavior habits for social media users.

Article 4 (Mensah et al.) focused on government information transparency and information adoption by analyzing how perceived government information transparency affected the adoption of information related to COVID-19 on social media based on the information adoption model. In this article, the authors examined the survey data collected from 516 participants. It was found that information quality, credibility, and usefulness effectively affected the adoption of COVID-19 pandemic information on social media. Meanwhile, the perceived government information transparency positively moderated the impact of information quality, credibility, and usefulness on adopting COVID-19 pandemic information on social media. This study shed light on combating the wave of false information, improving social media's ability to respond to public health events, and establishing a sound public health emergency response system.

Article 5 (Alon-Tirosh and Meir) used a qualitative approach and examined how a group of adolescents with autism spectrum disorders and difficulties in communication used social network sites to communicate. In this article, based on semi-structured interviews with ten adolescents diagnosed with autism spectrum disorder, the authors analyzed the reasons for using social networks, the specific situations of social network use, and the characteristics of autism reflected through social networking. The results provided insightful evidence about how social networks could play a role in the lives of adolescents with autism spectrum disorders, confirming that social media's social interaction function is even more essential for adolescents with autism.

## Author contributions

MC: Project administration, Writing—original draft, Writing—review and editing. PL: Project administration, Writing—original draft, Writing—review and editing. CX: Project administration, Writing—original draft, Writing—review and editing. HL: Writing—original draft.
